# Supplementary material for: Few SARS-CoV-2 infections detected in Newfoundland and Labrador in the absence of Public Health Laboratory-based confirmation
Source: PLoS One. 2022 Jan 28;17(1):e0262957. doi: 10.1371/journal.pone.0262957 (PMC8797227; doi:10.1371/journal.pone.0262957)
Supplement: Attachment — Submitted filename: ResponsetoReviewers.docx [file pone.0262957.s002.docx]

**Journal requirements:**

1. When submitting your revision, we need you to address these additional requirements.

Please ensure that your manuscript meets PLOS ONE's style requirements, including those for file naming. The PLOS ONE style templates can be found at 
<https://journals.plos.org/plosone/s/file?id=wjVg/PLOSOne_formatting_sample_main_body.pdf> and 
<https://journals.plos.org/plosone/s/file?id=ba62/PLOSOne_formatting_sample_title_authors_affiliations.pdf>

*The revised manuscript submitted has been altered to meet PLOS ONE’s style requirements.*

2. Please amend your current ethics statement to address the following concerns:
a) Did participants provide their written or verbal informed consent to participate in this study?
b) If consent was verbal, please explain i) why written consent was not obtained, ii) how you documented participant consent, and iii) whether the ethics committees/IRB approved this consent procedure.

*Our ethics statement has been revised to indicate that written informed consent was obtained from study participants. “*In accordance with the Declaration of Helsinki, written informed consent was obtained for whole blood collection at 3 month intervals and a questionnaire addressing previous testing history and reasons for suspecting infection with SARS-CoV-2 was administered at study intake.”, pg 6, line 127-130.

3. Thank you for stating the following financial disclosure: 
This work was supported by a Covid-19 rapid research funding opportunity grant (Funding Reference Number:  VR1 – 173202) from the Canadian Institutes for Health Research awarded through the COVID Immunity Task Force to MDG, RSR and KAH.

Please state what role the funders took in the study.  If the funders had no role, please state: The funders had no role in study design, data collection and analysis, decision to publish, or preparation of the manuscript." 
If this statement is not correct you must amend it as needed. 
Please include this amended Role of Funder statement in your cover letter; we will change the online submission form on your behalf.

*We have included the amended Role of Funder statement in our cover letter as requested. It reads “The funders had no role in study design, data collection and analysis, decision to publish, or preparation of the manuscript."*

4. In your Data Availability statement, you have not specified where the minimal data set underlying the results described in your manuscript can be found. PLOS defines a study's minimal data set as the underlying data used to reach the conclusions drawn in the manuscript and any additional data required to replicate the reported study findings in their entirety. All PLOS journals require that the minimal data set be made fully available. For more information about our data policy, please see <http://journals.plos.org/plosone/s/data-availability>.

Upon re-submitting your revised manuscript, please upload your study’s minimal underlying data set as either Supporting Information files or to a stable, public repository and include the relevant URLs, DOIs, or accession numbers within your revised cover letter. For a list of acceptable repositories, please see <http://journals.plos.org/plosone/s/data-availability#loc-recommended-repositories>. Any potentially identifying patient information must be fully anonymized.

Important: If there are ethical or legal restrictions to sharing your data publicly, please explain these restrictions in detail. Please see our guidelines for more information on what we consider unacceptable restrictions to publicly sharing data: <http://journals.plos.org/plosone/s/data-availability#loc-unacceptable-data-access-restrictions>. Note that it is not acceptable for the authors to be the sole named individuals responsible for ensuring data access.

We will update your Data Availability statement to reflect the information you provide in your cover letter.

*Minimal underlying data set up-loaded as “Supporting Information” as specified in cover letter*.

5. Thank you for stating the following in the Funding Section of your manuscript: 
"This work was supported by a Covid-19 rapid research funding opportunity grant (Funding Reference Number:  VR1 – 173202) from the Canadian Institutes for Health Research awarded through the COVID Immunity Task Force to MG, RR and KHo."

We note that you have provided funding information. However, funding information should not appear in the Funding section or other areas of your manuscript. We will only publish funding information present in the Funding Statement section of the online submission form. 
Please remove any funding-related text from the manuscript and let us know how you would like to update your Funding Statement. Currently, your Funding Statement reads as follows: 
"This work was supported by a Covid-19 rapid research funding opportunity grant (Funding Reference Number: VR1 – 173202) from the Canadian Institutes for Health Research awarded through the COVID Immunity Task Force to MG, RR and KHo"

Please include your amended statements within your cover letter; we will change the online submission form on your behalf.

*We have removed all funding-related text from the manuscript and included the original funding statement "This work was supported by a Covid-19 rapid research funding opportunity grant (Funding Reference Number: VR1 – 173202) from the Canadian Institutes for Health Research awarded through the COVID Immunity Task Force to MDG, RSR and KAH" in our cover letter for the online submission form.*

6. Please review your reference list to ensure that it is complete and correct. If you have cited papers that have been retracted, please include the rationale for doing so in the manuscript text, or remove these references and replace them with relevant current references. Any changes to the reference list should be mentioned in the rebuttal letter that accompanies your revised manuscript. If you need to cite a retracted article, indicate the article’s retracted status in the References list and also include a citation and full reference for the retraction notice.

*The only change to our reference list is inclusion of several new references in response to comments of the reviewers. Reference list is complete and correct.*

**Response to reviewers**

Reviewer # 1.

*Thank you for your comments and suggestions for improvement.*

1. Authors should provide limitations to their study.

*Several sentences addressing limitations of the study have been added to the discussion section of the text (lines -349-353)*

At least one additional Figure (illustration) may be provided as to highlight the summary or prospect of this study.

*We have added Figure 3, which shows the distribution of seropositive cases within the group of study subjects with PCR-confirmed SARS-CoV-2 infection and the group with unconfirmed, but suspected infection.*

3. To ensure the robustness and effectiveness of the risk model authors may support their finding with citing resent research articles to this manuscript.

*We have cited several additional recent research articles (references 11, 29-31) reinforcing the idea that the constant focus on a pandemic can feed anxiety and cause people to overestimate the likelihood of infection and to experience more severe symptoms.*

4. The abbreviations should be cross validated in the manuscript (First define them fully followed by abbreviation) and one paragraph can be added for abbreviations.

*Abbreviations have been full defined on first use and a paragraph listing abbreviations used was placed after the abstract as suggested by the reviewer.*

Reviewer #2:

*Thank you for your comments and suggestions for improvement.*

Comments:

1. The English of the manuscript can be polished.

*The manuscript has been read through thoroughly and altered as shown in track changes to improve the English.*

2. Line 219 in the manuscript looks misplaced.

The content of line 219 has been revised for clarity.

3. First two paragraphs of the discussion section can be shortened and relevant information about mortality rate and various prevention approaches should be provided related to immunity and health i.e. doi: 10.1007/s12088-020-00908-0 and information regarding anti-COVID-19 Agents DOI: 10.1007/s12088-020-00893-4 can be provided.

*The first 2 paragraphs of the discussion have been shortened slightly as suggested. The mortality rate for COVID-19 in the United States is present in reference 11. Prevention and treatment of COVID-19 is not a major focus of this manuscript, but reference to factors affecting risk for severe disease linked to new potential treatments are briefly discussed in lines 270-276 with new references 20-23 added.*

4. The title of the manuscript should be modified. The manuscript does not provide substantial proof that.

*The title has been modified to avoid creating the impression that unusually severe or persistent symptoms in persons in close contact with confirmed cases are due to rare SARS-CoV-2 variants.*

5. In conclusion, in lines 320 to 324, the authors signify selectivity for SAR-CoV-2 nucleocapsid in detecting previous exposure to SARS-CoV-2. However, in the following line, the author also mentions that detecting antibodies against SARS-CoV-2 nucleocapsid alone cannot be considered diagnostic of past COVID-19 infections. Please explain then how the idea of the study can be justified when these two claims contradict each other.

*We suggest that the presence of antibodies against the SARS-CoV-2 nucleocapsid cannot alone be considered indicative of past COVID-19 infection, because they may simply be cross-reactive antibodies induced by infection with common corona viruses. However, if there is selective binding of antibodies to the SARS-CoV-2 nucleocapsid over the nucleocapsids of common β-coronaviruses this can be considered indicative of past COVID-9 infection*.
6. In material methods section, the authors mention PBMCs are isolated and stored, but these PBMCs are not used in the manuscript. Therefore, please rewrite the material method section accordingly.

*The materials and methods section has been revised accordingly*.

7. The author has represented his results in optical density at 450 nm. However, a ratio-based analysis of samples can be done for precise representation

*The results are now represented with optical density at 450 nm normalized against the value of a standard concurrently run with each ELISA.*

8. Please mention how the cut-off value is determined for positivity based on reactivity with S or RBD in the manuscript.

*The cut-off value for positivity against S or RBD was determined using forty negative control plasma samples collected before October 2019. The cutoff was set at 2 standard deviations above the mean optical density at 450 nm for the 40 control samples. (lines 153-157).*
